# Pilot investigation of the rhizosphere microbial communities and metabolism of two cultivars of *Polygonatum cyrtonema* Hua

**DOI:** 10.3389/fmicb.2025.1615900

**Published:** 2025-07-23

**Authors:** Fang Liu, Wenlong Dong, Yi An, Hongyu Qian, Chunlin Gui, Yunjian Xu

**Affiliations:** ^1^School of Agriculture, Yunnan University, Kunming, China; ^2^Chizhou Rural Revitalization Industry Development Co., Ltd., Chizhou, China; ^3^Anhui Jinyunong Biotechnology Co., Ltd., Chizhou, China; ^4^State Key Laboratory for Vegetation Structure, Function and Construction (VegLab), Ministry of Education Key Laboratory for Transboundary Ecosecurity of Southwest China, and Yunnan Key Laboratory of Plant Reproductive Adaptation and Evolutionary Ecology, School of Ecology and Environmental Science, Institute of Biodiversity, Yunnan University, Kunming, China

**Keywords:** *Polygonatum cyrtonema* Hua, rhizosphere microbiota, fungal communities, metabolites, microbial-metabolite interactions

## Abstract

*Polygonatum cyrtonema* Hua, a medicinal herb valued in traditional Chinese medicine, produces bioactive polysaccharides and saponins, exhibits intraspecific metabolic variations whose interactions with rhizosphere microbiota remain unexplored. As a pilot investigation, we characterized these linkages in two representative high-yield cultivars (JH1: bead-like tubers; JH2: L-shaped rhizomes) through integrated 16S rRNA/ITS sequencing and metabolomics. Bacterial communities showed conserved composition (Proteobacteria-dominated; shared core genera *Candidatus Koribacter* and *Bradyrhizobium*), whereas fungal assemblages diverged sharply between cultivars. JH1 rhizospheres enriched *Hydnum*, *Collimyces*, *Ramariopsis* and *Coralloidiomyces*, whereas JH2 favored *Acremonium*, *Archaeospora*, *Didymosphaeria*, *Entoloma* and *Monacrosporium*. Metabolomic profiling revealed tissue-driven specialization as the primary determinant, with tubers accumulated oleoyl ethylamide/DL-malic acid and roots preferentially storing DL-arginine. The core bacteria exhibited consistent negative association with organ-specific metabolites, whereas, fungal interactions diverged. JH1 enriched fungi positively associated with tuber oleoyl ethylamide and root DL-arginine but negatively with tuber DL-malic acid, while JH2-enriched taxa showed inverse relationships. The enriched fungal communities (average positive correlation coefficient 0.39) demonstrated stronger tissue-specific metabolite coordination than bacteria (average positive correlation coefficient 0.15), suggesting potential mycobiome-mediated regulation of medicinal compound partitioning. This preliminary dissection of cultivar-associated microbial-metabolite interplay may provide a mechanistic framework for optimizing *P. cyrtonema* cultivation through synthetic microbial consortia. However, future multi-location, multi-season studies with soil controls are needed to validate ecological generality.

## Introduction

*Polygonatum cyrtonema* Hua, a perennial medicinal and edible herb of the Liliaceae family, is valued for its therapeutic and nutritional properties. Its bioactive compounds, including polysaccharides, saponins, flavonoids, and lignans, contribute to cardiovascular health, hypoglycemic effects, and antiaging activity (Wu et al., 2014; Yang et al., 2015). However, increasing reliance on wild harvesting has led to ecological strain and resource depletion, necessitating a shift to artificial cultivation. While cultivated production addresses market demand, it introduces challenges: expanding monoculture practices exacerbate soil degradation, disrupt rhizosphere microbial communities, and intensify disease susceptibility (e.g., root rot, anthracnose), ultimately compromising rhizome yield and medicinal quality (Chen et al., 2022; Zhou et al., 2017). These issues are further aggravated by autotoxicity, nutrient imbalances, and reduced bioactive compound synthesis under continuous cropping systems (Ji et al., 2023; Zeeshan Ul Haq et al., 2023), underscoring the need to optimize cultivation strategies by understanding cultivar-associated rhizosphere interactions.

Recent studies have highlighted complex relationships among the plant rhizosphere microbiota, root exudates, and medicinal plants health (Yuan et al., 2022). These findings show the systemic nature of rhizosphere interactions. Additionally, studies have found that microbial communities directly influence medicinal plant health by promoting growth, enhancing stress tolerance, and improving pathogen resistance (Castronovo et al., 2021; Hu et al., 2018; Shi et al., 2024; Tamang et al., 2024). Crucially, this microbial regulation appears bidirectional, as emerging evidence demonstrates that root exudate metabolites from medicinal plants significantly shape rhizosphere community composition and function (Kabir et al., 2024; Lyu et al., 2024). For example, root exudates of *P. cyrtonema* were found to recruit beneficial microbes that enhance plant defense against *Fusarium oxysporum* (Pang et al., 2022). However, while such these studies illuminate general plant-microbe-metabolite relationships, they primarily focus on single cultivar under controlled conditions. Therefore, this approach leaves a critical gap: it lacks insight into how genetic variations between cultivars influence divergent rhizosphere dynamics. This oversight is particularly important because previous study (Jia et al., 2025) has documented metabolic diversity among different cultivars. Consequently, the interactive mechanisms among plant genetic factors, root exudate profiles, and microbial community assembly in *P. cyrtonema* cultivars remain a key knowledge gap.

Our study represents a pilot investigation designed to systematically characterize potential cultivar-driven differences in the rhizosphere microbiome-metabolome interplay of *P. cyrtonema* and explore their possible implications for cultivation. By combination of 16S/ITS sequencing and LC-MS metabolomics, we analyzed microbial communities and root/rhizome metabolites across two dominant cultivars, and investigated their dynamic interactions. As an initial exploration, the integrated approach revealed preliminary associations suggesting cultivar-associated microbial consortia linked to distinct secondary metabolite profiles. Specifically, this work (1) identifies candidate key microbial taxa potentially relevant to *P. cyrtonema* growth and soil nutrient cycling, providing hypotheses for future biofertilizer development and cultivation protocol optimization, and (2) preliminarily explored the cultivar-associated metabolites, observing differential metabolites accumulation which potentially correlated with microbial community structures. These preliminary findings aim to stimulate mechanistic understanding of plant-microbe interactions in medicinal rhizosphere systems, and suggest a framework potentially applicable to other high-value Chinese herbs.

To better contextualize our study, it is important to acknowledge the significance of certain key microbes and metabolites involved. For instance, *Candidatus Koribacter* is known to play a role in soil nutrient cycling and plant growth promotion (Gómez-Godínez et al., 2025). *Bradyrhizobium* has been recognized for its nitrogen-fixing abilities and association with plant root systems, contributing to improved nutrient acquisition for plants (Roughley et al., 1995). Among the metabolites identified, DL-malic acid is involved in various physiological processes in plants, such as a source of carbon and energy and influencing microbial activities (Ke et al., 2018; Wei et al., 2021). The functional roles of oleoyl ethylamide in plants remain scarcely documented, whereas animal studies have demonstrated that this compound modulates energy metabolism and stress signaling pathways, concomitantly exerting anti-inflammatory and antioxidant activities (González-Portilla et al., 2025; Montagud-Romero et al., 2025; Tutunchi et al., 2023). Understanding the roles of these key microbes and metabolites and their relationships may inform future microbiome engineering strategies for enhancing bioactive compound production and may offer potential pathways for sustainable cultivation of medicinal plants worthy of further investigation.

## Materials and methods

### Rhizosphere soil and metabolite collection

**Figure 1:**
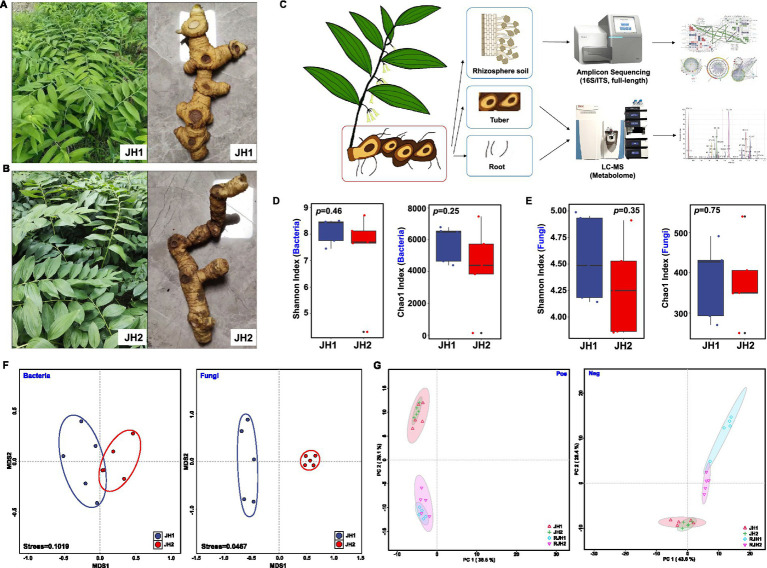
Comparison analysis of two *Polygonatum cyrtonema* Hua cultivars (JH1 and JH2). **(A)** Whole-plant and tuber morphology of JH1 cultivar. **(B)** Whole-plant and tuber morphology of JH2 cultivar. **(C)** Flow chart for the detection of fungi and bacteria in rhizosphere soil and LC-MS nontargeted metabolomic analysis of tubers and roots. **(D)** Shannon and Chao1 indices of bacterial alpha diversity. **(E)** Shannon and Chao1 indices of fungal alpha diversity. Boxes represent the interquartile range (IQR), and the whiskers extend to 1.5× IQR. Statistical significance was determined by Tukey’s HSD tests. **(F)** Beta diversity of bacteria and fungi in rhizosphere soil. **(G)** Principal component analysis (PCA) of metabolites from tubers (JH1 and JH2) and roots (RJH1 and RJH2) identified in positive ion mode (pos) and negative ion mode (neg).

To investigate cultivar-associated differences in rhizosphere microbial communities and root/rhizome metabolites, we selected two morphologically distinct *P. cyrtonema* cultivars (JH1 and JH2) from Jiuhua Mountain, Anhui Province, China, at 3-year-old. JH1 produces round, bead-like tubers, whereas JH2 develops elongated, L-shaped rhizomes (Figures 1A,B). The site features mountainous terrain with an elevation of 500–800 m, characterized by typical yellow-brown mountainous soil under a humid subtropical monsoon climate (mean annual temperature: 16°C; precipitation: 2,000 mm). The planting density was set at a row spacing of 40–50 cm and plant spacing of 30–40 cm, amounting to 6,900–7,400 plants per hectare under a forest-grown wild-simulated mode (understory planting). No fertilization was applied, in line with wild-simulated cultivation practices.

To collect rhizosphere soil samples, the roots and adhering soil were placed into a 50-mL centrifuge tube, and phosphate-buffered saline (PBS) was added. The tubes were then shaken on a platform for 20 min at 180 rpm to dislodge the soil (Beckers et al., 2017). After the roots were removed, the samples were centrifuged at 4,000 × g for 20 min at 4°C to separate and collect the rhizosphere soil (Edwards et al., 2015). Five replicate samples were prepared for each cultivar. Overall, 10 rhizosphere samples were collected. Rhizosphere soil and plant tissues (tuber and root) were sampled to concurrently analyze microbial community structure and root/rhizome metabolite profiles (Figure 1C). Paired sequencing workflows were used to characterize the microbiome and metabolome characterization.

### Metabolome analysis

For the investigation of root secondary metabolites, tuber and root samples were used. Metabolome sequencing was performed by Biozeron Biotech (Shanghai) Co., Ltd., China. For metabolome analysis, 100 mg of tissue was ground in liquid nitrogen and placed in an EP tube, followed by the addition of 500 μL of 80% methanol aqueous solution. The mixture was vortexed and then incubated on ice for 5 min. Subsequently, it was centrifuged at 15,000 × g for 20 min at 4°C. A portion of the supernatant was diluted with mass spectrometry (MS)-grade water to achieve a methanol concentration of 53%. The sample was then centrifuged again at 15,000 × g for 20 min at 4°C. The final supernatant was collected and injected into the UHPLC-MS/MS (Thermo Fisher, Germany) system analysis (Want et al., 2006). Five replicates were prepared for each sample type. A total of 20 metabolic samples were collected.

UHPLC-MS/MS analyses were performed using a Vanquish UHPLC system (Thermo Fisher, Germany) coupled with an Orbitrap Q ExactiveTM HF mass spectrometer (Thermo Fisher, Germany) in Biozeron Co., Ltd. (Shanghai, China). Samples were injected onto a Hypersil Gold column (100 × 2.1 mm, 1.9 μm) using a 17-min linear gradient at a flow rate of 0.2 mL/min. For positive polarity mode, the eluents were eluent A (0.1% formic acid in water) and eluent B (methanol). For negative polarity mode, the eluents were eluent A (5 mM ammonium acetate, pH 9.0) and eluent B (methanol). The solvent gradient was set as follows: 2% B for 1.5 min; linear increase to 100% B over 12.0 min; maintained at 100% B for 2.0 min; decreased to 2% B at 14.1 min; and held at 2% B until 17 min. The Q Exactive^™^ HF mass spectrometer was operated in positive/negative polarity mode with a spray voltage of 3.2 kV, capillary temperature of 320°C, sheath gas flow rate of 40 arb, and aux gas flow rate of 10 arb.

Raw mass spectrometry data were imported into Compound Discoverer 3.1 (CD3.1, Thermo Fisher Scientific). Initial filtering was applied to remove low-quality features based on retention time (RT) and mass-to-charge ratio (*m*/*z*). Peak alignment across samples was performed with a tolerance of 0.2 min for RT and 5 ppm for *m*/*z*. Feature extraction was conducted with parameters set to 5 ppm mass tolerance, 30% intensity tolerance, signal-to-noise ratio ≥3, and minimum intensity threshold. Metabolite identification was achieved by matching experimental MS1 spectra against the mzCloud and mzVault databases and MS2 spectra against MassList. For relative quantification, peak areas were integrated, and raw data were normalized using total ion current (TIC) normalization to correct for injection variability. Quality control (QC) samples were used to filter metabolites with a coefficient of variance (CV) >30%, ensuring reproducibility. Final normalized data were log2-transformed to approximate normal distribution for downstream statistical analyses.

### Bacterial and fungal amplicon sequencing and analysis

The total DNA of the rhizosphere soil was extracted via a PowerSoil^®^ DNA isolation kit (Mo Bio, Laboratories Inc., United States) according to the manufacturer’s instructions. The primers used to amplify 16S ribosomal DNA (rDNA) were 27F and 1492R (Defez et al., 2017), while the primers used to amplify fungal ITS fragments were ITS1F and ITS4R (White et al., 1990). PCR reactions were performed in triplicate 20 μL mixture containing 4 μL of 5 × FastPfu Buffer, 2 μL of 2.5 mM dNTPs, 0.8 μL of each primer (5 μM), 0.4 μL of FastPfu Polymerase, and 10 ng of template DNA. Amplicons were extracted from 2% agarose gels and purified using the AxyPrep DNA Gel Extraction Kit (Axygen Biosciences, Union City, CA, United States) according to the manufacturer’s instructions. Sequencing was performed on the PacBio Sequel IIe platform by Sangon Biotech (Shanghai) Co., Ltd., China.

Raw reads were processed using the SMRT Link Analysis software (version 11.0) to generate demultiplexed circular consensus sequence (CCS) reads, with settings including a minimum of 3 passes and a minimum predicted accuracy of 0.99. Barcode and primer sequences were removed using the lima pipeline (Pacific Biosciences demultiplexing barcoded software, https://lima.how/). Quality control, assembly, and merging of sequencing data into original amplicon sequence variants (ASVs) were performed using the DADA2 package in R (version 3.6.0) (Callahan et al., 2016). The UCHIME method was employed for the identification and removal of chimeric sequences to ensure the accuracy of subsequent analyses. Taxonomic classification of bacteria and fungi was conducted using the SILVA database (version 13.2) (Quast et al., 2013) and the UNITE database (version 8.0), respectively. Sequences annotated as “Mitochondria” (taxonomic path: k__Eukaryota; c__Mitochondria) or “Chloroplast” were removed using the taxa_remove_taxa() function in the R package phyloseq. To further ensure completeness, we aligned ASV sequences against the host plant’s mitochondrial and chloroplast genomes using VSEARCH with a similarity threshold of ≥97%. Any ASVs matching these references were excluded. Post-filtering, we verified the effectiveness of contamination removal by quantifying the residual mitochondrial and chloroplast reads, which accounted for <0.1% of the total filtered reads. DESeq2 was used to identify differentially abundant taxa with default parameters (Love et al., 2014; Mandal et al., 2015) (Supplementary Tables S1, S2). For 16S rRNA rhizosphere sequencing, samples JH1 and JH2 yielded 33,199 and 33,084 high-quality sequences respectively, with average lengths of 1,442 bp and 1,445 bp. ITS sequencing generated 40,988 (JH1) and 57,329 (JH2) sequences, averaging 591 bp and 580 bp. The raw sequencing data have been submitted to NCBI under the project accession code PRJNA1275732.

For the measurements of alpha diversity, Shannon and Chao1 indices were calculated to assess the species richness and evenness within the samples. Data normality and variance homogeneity were assessed using Shapiro–Wilk (*α* = 0.05) and Levene’s tests (*α* = 0.05), respectively. For group comparisons, Student’s *t*-test (parametric) or Mann–Whitney *U* test (non-parametric) was used, with effect sizes calculated as Cohen’s *d* (*t*-tests) or rank-biserial correlation (Mann–Whitney), the beta diversity of microbes was performed using non-metric multidimensional scaling (NMDS) with the vegan community ecology package, R-forge.^1^ Community structural differences were assessed using Bray–Curtis dissimilarity matrices. Permutational multivariate analysis of variance (PERMANOVA) was performed with adonis2 (vegan v2.6-4; 9999 permutations). Where significant differences were detected (*p* < 0.05), pairwise PERMANOVA tests were applied to identify specific group contrasts. Complementary analyses (ANOSIM, MRPP) were retained for methodological consistency with prior literature (Wei et al., 2019). Core bacterial taxa identified as the intersection of rhizosphere bacterial communities from both cultivars, with abundances ranking in the top 5 of all bacterial taxa. Core fungal taxa exhibiting significant upregulation in both cultivars (FDR <0.05) and ranking within the top 25 of fungal taxa by abundance.

To predict the functional profiles of bacterial and fungal communities in different samples, Phylogenetic Investigation of Communities by Reconstruction of Unobserved States (PICRUSt2)^2^ and FUNGuild were employed. PICRUSt2 was used to predict the functional profiles based on the Kyoto Encyclopedia of Genes and Genomes (KEGG) database (Langille et al., 2013), while FUNGuild was used to predict the ecological guilds of fungi based on their taxonomic affiliations. While these results suggest potential metabolic contributions, they require validation via metatranscriptomics or enzyme assays. Co-occurrence networks were constructed using the WGCNA package based on Spearman’s correlation matrices (Spearman *r* > 0.8, *p*-value <0.05) of amplicon sequencing data. Only ASVs with relative abundance >0.01% and detection rate >60% across samples were retained for analysis. The igraph package was used for visualization of network diagrams. In the networks, each ASV was represented as a node, while significant correlations between ASVs were visualized as edges. The redundancy analysis (RDA) was employed to examine the relationships between the relative abundance of microbial communities at the genera levels and metabolites. Linear regression and Pearson correlation analyses were performed using JMP 10.0 (SAS Institute, Cary, NC, United States). The RDA was conducted using the R statistical environment (v3.6.3; http://www.r-project.org/).

## Results

### Cultivar-associated rhizosphere microbiome assembly and tissue-determined metabolic profiling in *Polygonatum cyrtonema*

Comparative analysis of rhizosphere microbiota revealed divergent assembly patterns between cultivars. Bacterial alpha diversity showed no significant cultivar differences in evenness (Shannon; Mann–Whitney *U* = 10.0, *p* > 0.05, *r* = 0.23) or richness (Chao1; *U* = 12.0, *p* > 0.05, *r* = 0.36) (Figure 1D). Similarly, fungal evenness (Shannon; *t* = 0.97, *p* > 0.05, *d* = 0.61) and richness (Chao1; *U* = 14.0, *p* > 0.05, *r* = 0.10) were statistically indistinguishable (Figure 1E). However, beta diversity analyses uncovered distinct community structuring. Nonmetric dimensional scaling (NMDS) analysis suggested partial overlap in bacterial communities (stress = 0.10), whereas, fungal assemblages showed complete separation by cultivar (stress = 0.05) (Figure 1F). PERMANOVA based on Bray–Curtis dissimilarity confirmed significant cultivar differences in fungal communities (*F* = 8.692, *R*^2^ = 0.491, *p* = 0.001) but not in bacterial communities (*F* = 1.447, *R*^2^ = 0.138, *p* = 0.185). Complementing microbial data, untargeted metabolomics of root/tuber extracts identified 2,190 metabolites (Supplementary Table S3). Principal component analysis (PCA) suggested organ-dependent metabolic differentiation as the dominant trend. While root-tuber comparisons showed pronounced compositional shifts (Pos PC2: 29.1% variance; Neg PC2: 26.4% variance), inter-cultivar differences within the same organ were minimal (Pos PC2: 38.5% variance; Neg PC2: 43.6% variance; Figure 1G).

### Microbial community composition in different cultivars of *Polygonatum cyrtonema*

**Figure 2:**
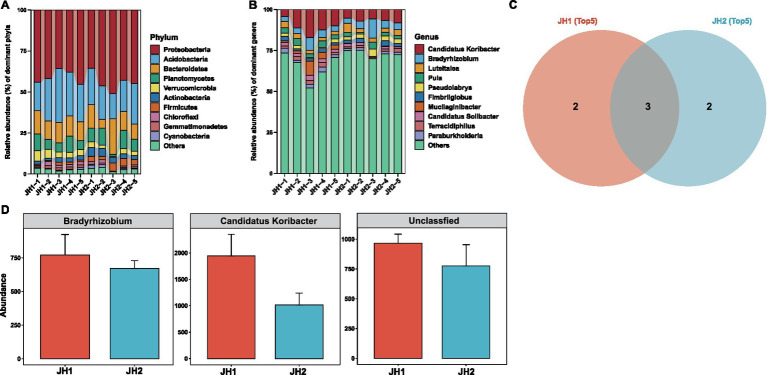
Composition and function of the rhizosphere bacterial communities of two *Polygonatum cyrtonema* Hua cultivars. Bacterial community composition at the phylum level **(A)** and genus level **(B)**. **(C)** The Venn diagram illustrates the distribution of the top 5 significantly enriched bacterial genera in JH1 and JH2 samples, respectively. The diagram clearly divides into three regions: the area representing bacterial genera exclusively enriched in JH1 (pink), the area representing bacterial genera exclusively enriched in JH2 (blue), and the intersecting region representing enriched genera shared by both groups (shared genera). **(D)** Abundances of members of the top 5 most abundant genera that are shared between JH1 and JH2.

Bacterial community composition. To elucidate differences in microbial communities between the two cultivars, we first analyzed the rhizosphere bacterial composition of JH1 and JH2. The bacterial abundance at both phylum and genus levels exhibited remarkable similarity between the two cultivars (Figures 2A,B). Proteobacteria dominated at the phylum level, while *Candidatus Koribacter* emerged as the most abundant genus in both cultivars. Further analysis revealed that JH1 and JH2 shared two of the top five most abundant bacterial genera: *Candidatus Koribacter* (8.74 and 5.63%) and *Bradyrhizobium* (3.47 and 3.73%) (Figure 2C). Intriguingly, these shared genera demonstrated higher relative abundances in the rhizosphere of JH1 compared to JH2 (Figure 2D). Functional predictions suggested key metabolic pathways associated with bacterial communities, including chemoheterotrophy, aerobic chemoheterotrophy, nitrate reduction, and ureolysis, with no significant functional divergence observed between the cultivars (Supplementary Figure S1A). These findings collectively suggest a conserved bacterial community structure in the rhizospheres of JH1 and JH2.

**Figure 3:**
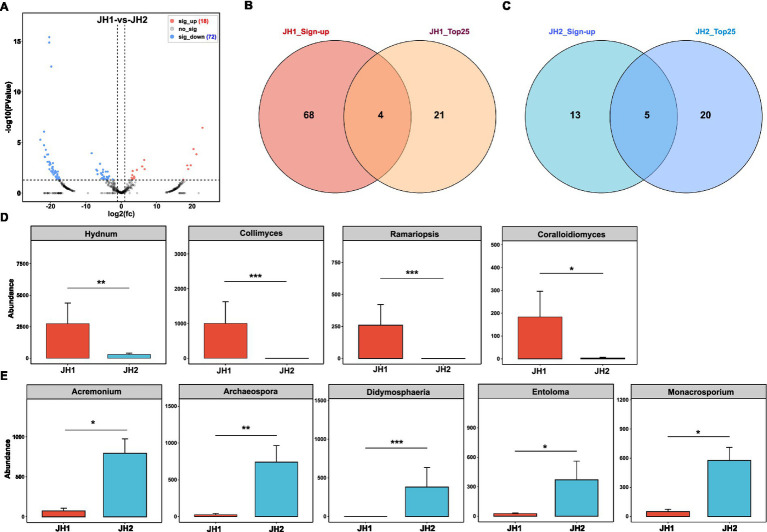
Rhizosphere fungal community composition of two *Polygonatum cyrtonema* Hua cultivars. **(A)** Differences in fungal community composition between JH1 and JH2 cultivars at the genus level. Venn diagram showing overlap between significantly upregulated and top 25 enriched fungal genera in JH1 **(B)** and in JH2 **(C)**. **(D)** Abundances of members of the top 25 fungal genera that are present in JH1. **(E)** Abundances of members of the top 25 fungal genera that are present in JH2.

Fungal community composition. In contrast to the bacterial communities, fungal composition diverged substantially between the two cultivars (Figure 3A). Comparative analysis identified 72 fungal genera enriched in JH2 and 18 genera enriched in JH1. Among the top 25 most abundant fungal genera, JH1 exhibited elevated abundances of *Hydnum*, *Collimyces*, *Ramariopsis*, and *Coralloidiomyces* (Figure 3B), whereas JH2 showed enrichment in *Acremonium*, *Archaeospora*, *Didymosphaeria*, *Entoloma*, and *Monacrosporium* (Figure 3C). Statistical validation using Mann–Whitney *U* tests (with Benjamini–Hochberg correction) confirmed significant increases in *Hydnum* [8.34% vs. 0.65%; *U* = 0.0, *p* = 0.008, *r* = 0.84, 95% CI (6.82, 9.15)], *Collimyces* Collimyces [3.04% vs. 0.01%; *U* = 1.0, *p* = 0.009, *r* = 0.82, 95% CI (2.68, 3.27)], *Ramariopsis* [0.79% vs. 0; *U* = 0.0, *p* = 0.008, *r* = 0.84, 95% CI (0.71, 0.85)], and *Coralloidiomyces* [0.56% vs. 0; *U* = 0.0, *p* = 0.008, *r* = 0.84, 95% CI (0.49, 0.61)] in JH1 relative to JH2 (Figure 3D), alongside marked reductions in *Acremonium* [0.23% vs. 1.80%; *U* = 0.0, *p* = 0.008, *r* = 0.84, 95% CI (−1.58, −0.92)], *Archaeospora* [0.08% vs. 1.68%; *U* = 0.0, *p* = 0.008, *r* = 0.84, 95% CI (−1.52, −0.78)], *Didymosphaeria* [0.00% vs. 0.87%; *U* = 0.0, *p* = 0.008, *r* = 0.84, 95% CI (−0.85, −0.72)], *Entoloma* [0.08% vs. 0.84%; *U* = 0.0, *p* = 0.008, *r* = 0.84, 95% CI (−0.76, −0.68)], and *Monacrosporium* [0.15% vs. 1.31%; *U* = 0.0, *p* = 0.008, *r* = 0.84, 95% CI (−1.18, −0.91)] in JH1 (Figure 3E).

Functional prediction of fungi suggested a diverse array of potential functional roles for the fungal taxa identified in the rhizosphere samples (Supplementary Figure S1B). Guild assignment indicated dominant representation in mutualistic and decomposer communities: symbiotic nutrition (Arbuscular mycorrhizal and Ericoid mycorrhizal) constituted the core mutualists, potentially supporting plant-fungal partnerships for nutrient acquisition; decomposition pathways (wood saprotrophs and undefined saprotrophs) dominated nutrient cycling profiles, consistent with lignocellulose degradation in root detritus-rich environments; host-associated interactions (root-associated biotrophs and epiphytes) suggested specialized root surface colonization strategies; parasitic interactions were evidenced by fungal parasites and lichen parasites. This functional partitioning characterizes the rhizosphere as a multitrophic interface where nutrient mobilization (saprotrophs), plant symbiosis (mycorrhizae), and pathogenic constraints coexist, with potential implications for *P. cyrtonema* fitness.

To further explore microbial interactions, co-occurrence networks for bacteria and fungi in the rhizosphere of JH1 and JH2 were constructed independently (Supplementary Figures S2A–D). The bacterial network in JH1 exhibited lower taxonomic complexity, with nodes representing only four phyla compared to 11 phyla in JH2 (Supplementary Figures S2A,B). In contrast, fungal networks in both cultivars encompassed an identical number of phyla (five each). However, the JH2 fungal network demonstrated substantially higher connectivity, with more co-occurrence links than JH1 (Supplementary Figures S2C,D). These results indicate that microbial associations within the JH2 rhizosphere were more complex than those in JH1.

### Distinct metabolite profiles and core microbial correlations in JH1 and JH2 cultivars

**Figure 4:**
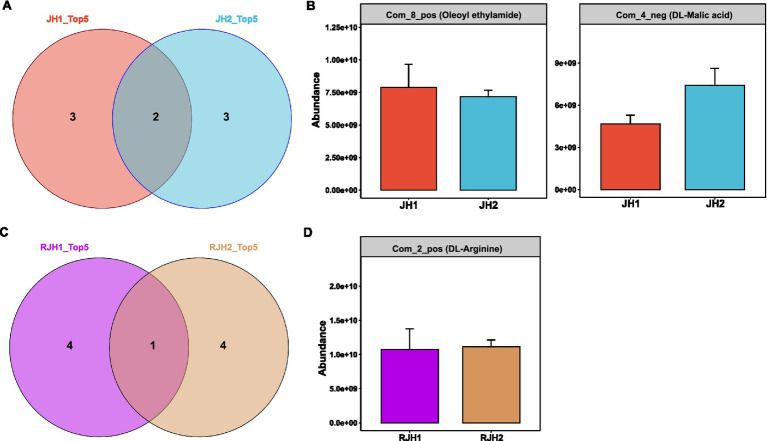
Comparison of metabolites from the tubers and roots of two *Polygonatum cyrtonema* Hua cultivars. **(A)** Venn diagram showing the overlap of the top 5 tuber metabolites between JH1 and JH2 cultivars. **(B)** Abundances of tuber metabolites shared between JH1 and JH2 cultivars. **(C)** Venn diagram showing the overlap of the top 5 root metabolites between JH1 and JH2 cultivars. **(D)** Abundances of root metabolites shared between JH1 and JH2 cultivars.

To investigate tissue-specific interactions between microbial communities and metabolites in JH1 and JH2, we analyzed root and tuber exudates. Among the top five tuber metabolites by abundance, both cultivars shared two compounds: Com_8_pos (oleoyl ethylamide) and Com_4_neg (DL-malic acid), while each exhibited three unique metabolites (Figure 4A). Quantitatively, the levels of oleoyl ethylamide and DL-malic acid showed no significant differences between JH1 and JH2 (Figure 4B). For root exudates, only one metabolite, Com_2_pos (DL-arginine), was common between cultivars (Figure 4C), with comparable relative abundance observed (Figure 4D). These findings highlight tissue-specific metabolic signatures, with limited overlap in root exudates compared to tubers.

**Figure 5:**
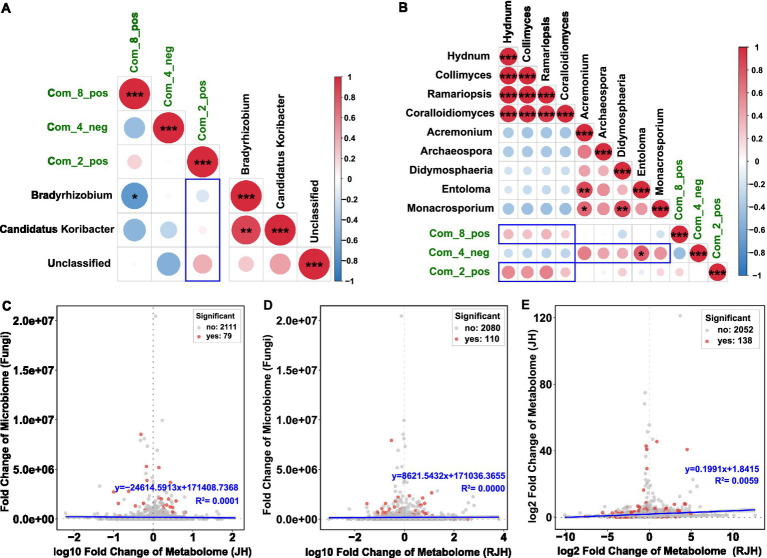
Correlations between metabolites and microorganisms. **(A)** Heatmap showing associations between specific metabolites and core bacterial communities. **(B)** Heatmap showing associations between specific metabolites and core fungal communities. The depth of color represents the magnitude of the correlation, with blue indicating a negative correlation and red indicating a positive correlation. Correlation analysis was performed using Spearman’s method (^*^p < 0.05, ^**^p < 0.01, and ^***^p < 0.001). **(C)** Scatter plots of the relationships between tube metabolites and rhizosphere fungi. **(D)** Scatter plots of the relationships between root metabolites and rhizosphere fungi. **(E)** Scatter plots of the relationships between tuber metabolites and root metabolites.

Based on metabolite profiling, we conducted Spearman’s correlation analysis to investigate functional links between key microbial taxa and tissue-specific metabolites. Three bacterial genera (*Candidatus Koribacter*, *Bradyrhizobium*, and *Unclassified*) showed consistent negative associations with two tuber metabolites (oleoyl ethylamide and DL-malic acid) (Figure 5A and Supplementary Table S4). Among them, *Bradyrhizobium* showed a significant negative correlations with oleoyl ethylamide [*r* = −0.725, 95% CI (−0.930, −0.176), *p* = 0.018]. The associations between root-derived DL-arginine and these bacterial taxa exhibited distinct patterns: *Bradyrhizobium* showed a negative trend; *Candidatus Koribacter* displayed negligible association; *Unclassified* exhibited a weak positive correlation. Fungal correlations highlighted tissue specificity.

Key fungi enriched in JH1 (*Hydnum*, *Collimyces*, *Ramariopsis* and *Coralloidiomyces*) showed positive associations with oleoyl ethylamide and DL-arginine, but negative associations with DL-malic acid (Figure 5B, Supplementary Table S5). In JH2, dominant fungi (*Acremonium*, *Archaeospora*, *Didymosphaeria*, *Entoloma* and *Monacrosporium*) showed opposite patterns: negative associations with oleoyl ethylamide and DL-arginine, and associations with DL-malic acid. Correlation fitting analysis demonstrated three significant associations: rhizosphere fungal communities with tuber metabolites (Figure 5C); rhizosphere fungal communities with root metabolites (Figure 5D), and tuber metabolites with root metabolites (Figure 5E).

## Discussion

This study aimed to elucidate host genetic variation in two *Polygonatum cyrtonema* cultivars may influence rhizosphere microbial communities and root/tuber metabolite profiles. This dual perspective is crucial for understanding plant-microbe-metabolite interplay, particularly in characterizing cultivar-driven differences in microbiome-metabolome interactions. By comparing morphologically distinct cultivars (JH1 and JH2), the present analysis suggests three major observations: (1) Cultivar-associated effects appears to exert stronger selection on fungal communities than on bacterial assemblages, although bacterial functional profiles exhibit conservation; (2) Root/tuber metabolic signatures tend to be predominantly tissue-specific, with cultivar-driven differentiation being relatively limited, a pattern that stands in contrast to the observed fungal niche specialization; (3) Fungal taxa exhibit compartmentalized, tissue-dependent correlations with metabolites, which may imply their dominance in mediating plant-microbe metabolic crosstalk.

Consistent with studies on ginseng and rice cultivars (Volpe et al., 2023; Wang et al., 2021), we observed fungal communities to be more responsive to host genetic variation than bacteria. While bacterial α-/β-diversity and functional profiles (e.g., chemoheterotrophy, nitrate reduction) remained conserved across cultivars, fungal assemblages diverged markedly. These results suggested a pronounced cultivar-associated differentiation in fungal community structure. This disparity aligns with emerging evidence that fungi exhibit stronger host specificity through specialized symbiotic strategies (Volpe et al., 2023). Specifically, this study identified *Hydnum* and *Collimyces* were enriched in JH1, while *Acremonium* and *Didymosphaeria* were enriched in in JH2. Previous studies have associated *Hydnum* and *Collimyces* with organic acid metabolism, while *Acremonium* and *Didymosphaeria* have been identified as genera containing stress-tolerant endophytes (Marrassini et al., 2024; Ren et al., 2022). These findings suggest that JH1-enriched *Hydnum* and *Collimyces* may modulate organic acid metabolism, potentially influencing its tuber morphology. Conversely, JH2’s enrichment of stress-tolerant *Acremonium* and *Didymosphaeri*a could facilitate adaptive rhizome elongation in resource-limited soils by enhancing stress resilience. Crucially, our work extends prior observations by demonstrating that functional redundancy in bacterial communities (e.g., shared dominance of *Candidatus Koribacter* and *Bradyrhizobium*) buffers against host cultivars, whereas fungal guilds reflect cultivar-associated selection-a novel insight with implications for microbiome engineering in medicinal plants.

Metabolomic analysis revealed that tissue type was the primary driver of metabolite composition, outweighing cultivar effects. Roots exhibited DL-arginine dominance, which promotes root architecture through polyamine-mediated cell division via ornithine decarboxylase activation and nitric oxide biosynthesis enhancing lateral root initiation (Li et al., 2024; Wang et al., 2010; Wimalasekera and Scherer, 2022). Tubers accumulated DL-malic acid conferring stress, such as chelating rhizotoxic aluminum via malate-Al^3+^ complexation in acidic soils and sustaining redox homeostasis through NADPH regeneration in the GABA shunt (Kochian et al., 2015; Michaeli et al., 2011; Yuan et al., 2023). This metabolic conservatism prioritizes organ-specific physiology over cultivar adaptation. These metabolic constraints operate orthogonally to the observed fungal niche specialization, highlighting divergent evolutionary strategies in plant-microbe interactions.

The results of the correlation analysis between rhizosphere microbes and metabolites provide further evidence of the complex relationships between the microbial community and plant metabolism. Integrating microbiome-metabolome correlations revealed fungi as key mediators of tissue-specific metabolic dynamics. JH1-enriched fungi (*Hydnum*, *Ramariopsis*) correlated positively with tuber oleoyl ethylamide and root DL-arginine but negatively with tuber DL-malic acid, suggesting roles in lipid signaling and nutrient mobilization. Conversely, JH2-associated fungi (*Acremonium*, *Monacrosporium*) showed inverse trends. Strikingly, bacterial correlations were weaker and functionally ambiguous, reinforcing the primacy of fungi in mediating host metabolic crosstalk. This compartmentalized synergy implies that fungal symbionts may influence metabolite fluxes to match organ-specific demands, a mechanistic hypothesis need validation via targeted metabolite tracing.

While our dual-omics approach provides novel insights, some limitations warrant consideration. First, the study focused on two cultivars from a single geographic region; expanding to diverse ecotypes would clarify the generality of observed patterns. Second, correlative links between microbes and metabolites require functional validation, for example, synthetic community experiments to test causality. Future studies should integrate transcriptomics to resolve how host genetic networks interface with microbial and metabolic dynamics across tissues. Beyond advancing understanding of *P. cyrtonema* ecology, our findings offer a framework for optimizing medicinal plant cultivation. The decoupling of fungal niche specialization (cultivar-associated) and metabolic conservatism (tissue-driven) suggests that breeding programs targeting microbiome-enhanced resilience could focus on fungal symbiont selection without disrupting core metabolic traits. Furthermore, the tissue-specificity of microbial-metabolite networks suggest the need for organ-resolved microbiome management in precision agriculture.

While our integrated metabolomic and microbiome analysis provides novel insights into the cultivar-associated microbial associations and metabolite partitioning in *P. cyrtonema*, several design limitations warrant consideration when interpreting the results and highlight avenues for future research. Firstly, our study focused on only two high-yielding cultivars (JH1 and JH2), which limits the statistical power for broader inferences across the species’ diversity and precludes generalization to other cultivars. Secondly, the study was conducted at a single geographical location. Consequently, the observed differences in fungal communities and metabolite profiles between JH1 and JH2 could potentially be confounded by unmeasured location-specific environmental factors, making it difficult to definitively separate cultivar-associated effects from environmental influences. Thirdly, our sampling represents a single time point, lacking temporal replication across seasons or developmental stages. This prevents us from assessing potential seasonal or developmental dynamics in the rhizosphere microbiome-metabolite interactions. Finally, the absence of bulk soil controls analyzed alongside the rhizosphere samples limits our ability to definitively distinguish rhizosphere-specific enrichment or depletion patterns from the background soil microbial community. Future studies incorporating multiple cultivars across diverse locations, longitudinal sampling, and bulk soil controls will be essential to robustly validate the cultivar-associated microbial associations and their roles in regulating bioactive compound accumulation identified here, and to fully disentangle genetic, environmental, and temporal contributions to the rhizosphere metabolome-microbiome nexus in *P. cyrtonema*.

## Conclusion

This study establishes the mechanistic linkages between cultivar-associated rhizosphere microbiomes and tissue-partitioned medicinal metabolites in *Polygonatum cyrtonema*. While bacterial communities remained conserved across cultivars (JH1 and JH2), fungal assemblages diverged significantly, exhibiting cultivar-enriched taxa that correlated strongly with tissue-specific metabolite accumulation. Critically, fungal communities demonstrated superior coordination with medicinal compound partitioning (e.g., oleoyl ethylamide in tubers, DL-arginine in roots) compared to bacteria, implicating the mycobiome may be a key regulator of bioactive compound biosynthesis. These findings potentially provide a targeted roadmap for enhancing medicinal yields through synthetic microbial consortia, with fungal community engineering emerging as a strategic priority. Given the pilot-scale design with two cultivars, findings should be interpreted as hypothesis-generating rather than definitive. Additionally, while these mechanisms may offer insights for other rhizomatous crops, their applicability likely depends on species-specific traits such as root exudate profiles, microbial recruitment strategies, and stress response pathways. Further validation across diverse taxa is warranted. Future studies should incorporate multi-season, multi-location trials with expanded cultivar diversity and bulk soil controls to fully resolve cultivar-environment-metabolite interactions.

## Data Availability

The original contributions presented in the study are included in the article/Supplementary material, further inquiries can be directed to the corresponding authors.

## Footnotes

^1^ https://r-forge.r-project.org/

^2^ http://picrust.github.io/picrust/tutorials/genome_prediction.html

## Supplementary material

SUPPLEMENTARY FIGURE S1 Functional analysis of rhizosphere bacteria **(A)** and fungi **(B)** in JH1 and JH2 cultivars.

SUPPLEMENTARY FIGURE S2 Co-occurrence network analysis. Each node was labelled at the phylum level. **(A)** Co-occurrence network analysis for rhizosphere bacteria in the JH1. **(B)** Co-occurrence network analysis for rhizosphere bacteria in the JH2. **(C)** Co-occurrence network analysis for rhizosphere fungi in the JH1. **(D)** Co-occurrence network analysis for rhizosphere fungi in the JH2. A connection stands for a strong (Spearman’s *p* > 0.6) and significant (*p* < 0.05) correlation. The size of each node is proportional to the relative abundance.
