# Supplementary figures and images for: Pilot investigation of the rhizosphere microbial communities and metabolism of two cultivars of Polygonatum cyrtonema Hua
Source: Front Microbiol. 2025 Jul 23;16:1615900. doi: 10.3389/fmicb.2025.1615900 (PMC12325395; doi:10.3389/fmicb.2025.1615900)

**A**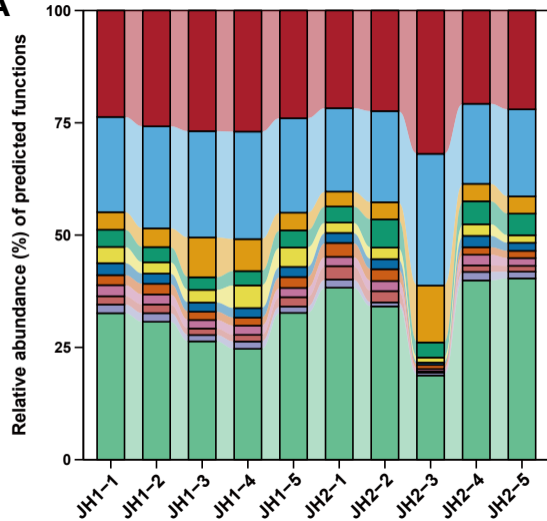**B**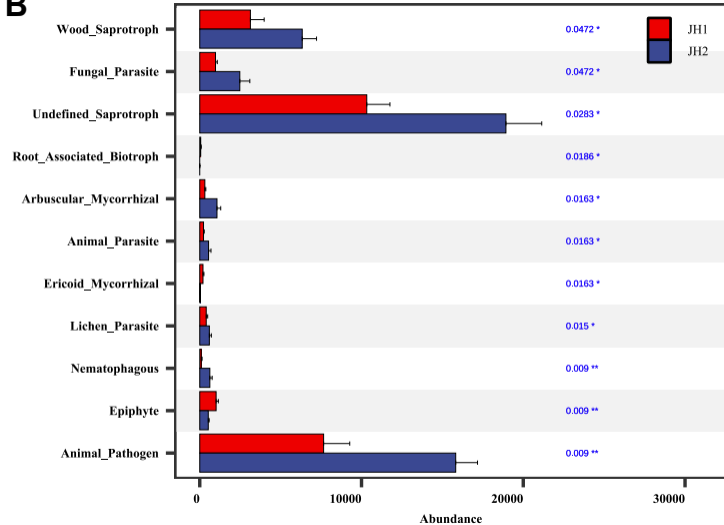

Supplement: SUPPLEMENTARY FIGURE S1 — Functional analysis of rhizosphere bacteria (A) and fungi (B) in JH1 and JH2 cultivars. [file Image_1.PDF]

**A**

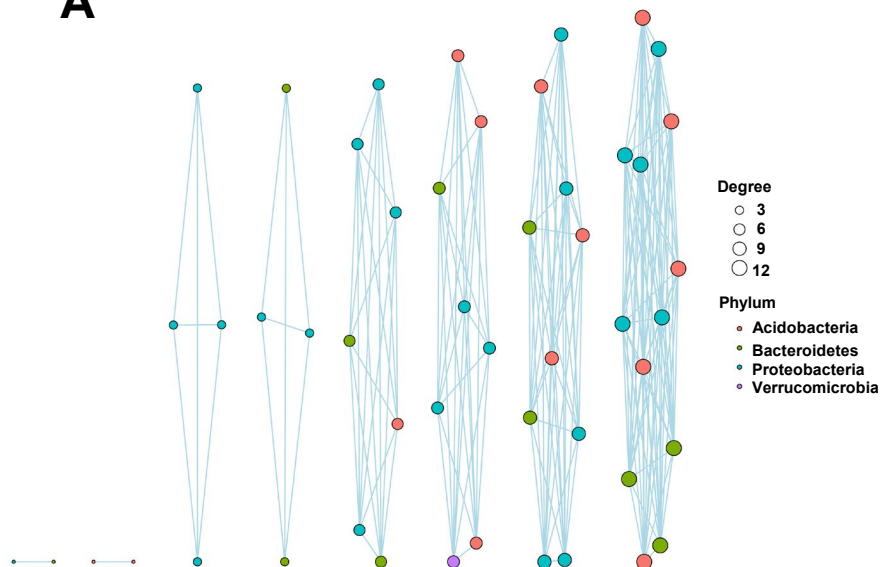

**B**

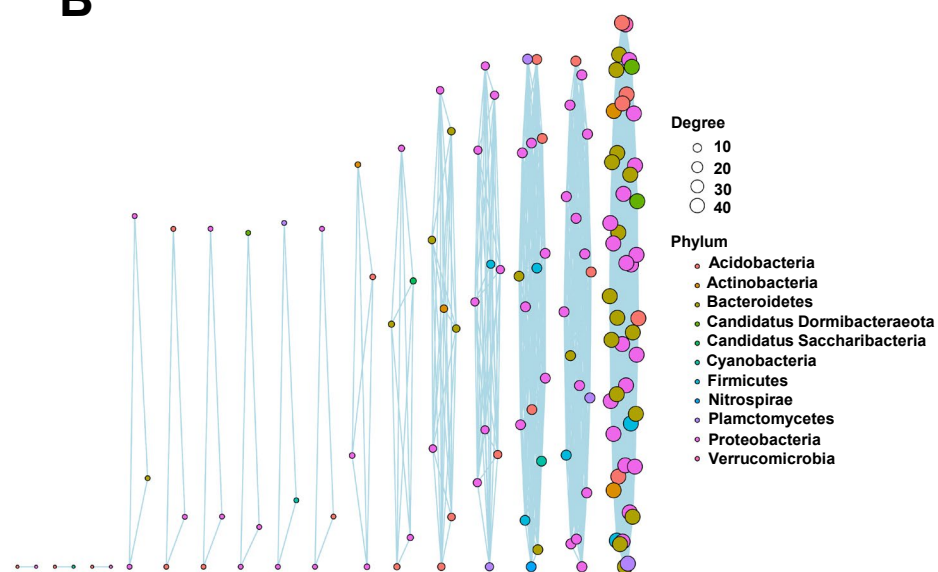

**C**

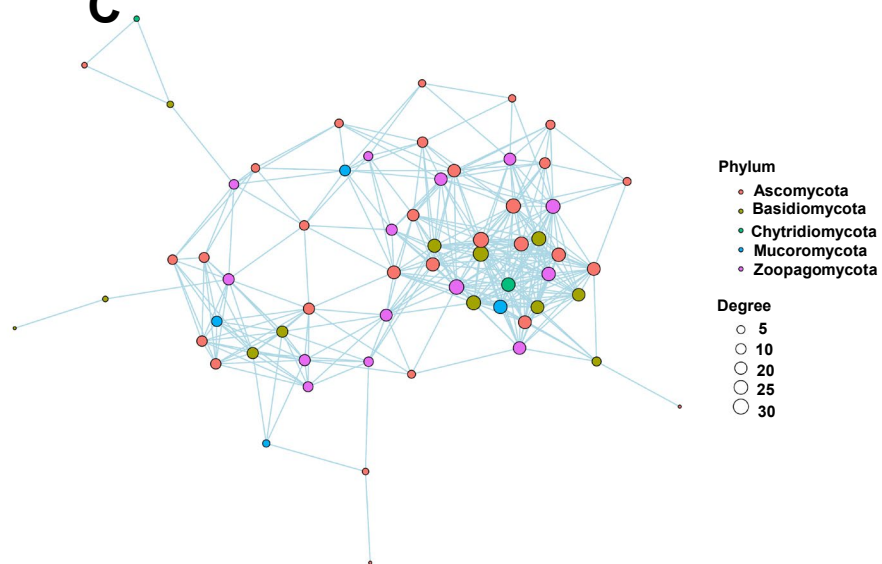

**D**

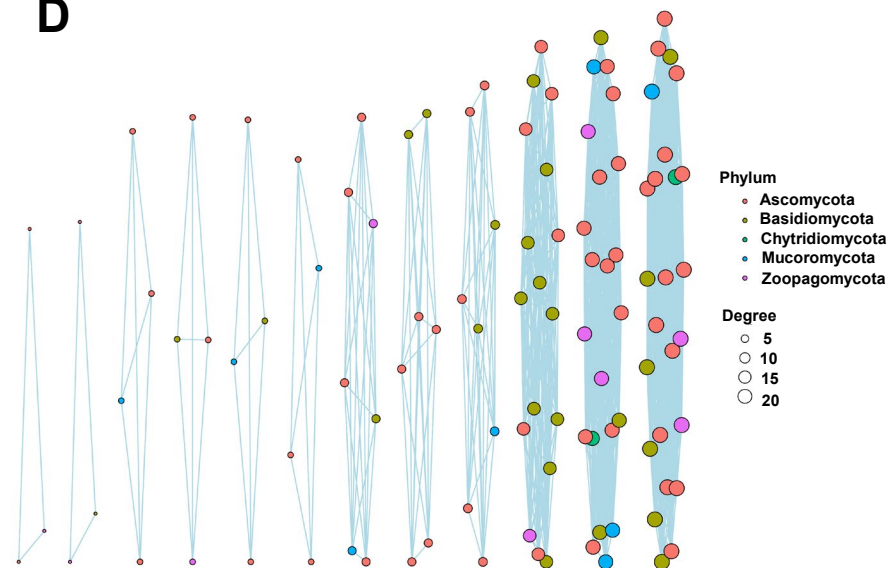

Supplement: SUPPLEMENTARY FIGURE S2 — Co-occurrence network analysis. Each node was labelled at the phylum level. (A) Co-occurrence network analysis for rhizosphere bacteria in the JH1. (B) Co-occurrence network analysis for rhizosphere bacteria in the JH2. (C) Co-occurrence network analysis for rhizosphere fungi in the JH1. (D) Co-occurrence network analysis for rhizosphere fungi in the JH2. A connection stands for a strong (Spearman’s p > 0.6) and significant (p < 0.05) correlation. The size of each node is proportional to the relative abundance. [file Image_2.PDF]
